# Evaluation of interventions for informed consent for randomised controlled trials (ELICIT): protocol for a systematic review of the literature and identification of a core outcome set using a Delphi survey

**DOI:** 10.1186/s13063-015-1011-8

**Published:** 2015-10-27

**Authors:** Katie Gillies, Vikki Entwistle, Shaun P. Treweek, Cynthia Fraser, Paula R. Williamson, Marion K. Campbell

**Affiliations:** Health Services Research Unit, University of Aberdeen, Health Sciences Building, Foresterhill, Aberdeen, AB25 2ZB UK; Department of Biostatistics, University of Liverpool, Shelley’s Cottage, Brownlow Street, Liverpool, L69 3GS UK

**Keywords:** Core outcome set, consensus methods, stakeholders, informed consent

## Abstract

**Background:**

The process of obtaining informed consent for participation in randomised controlled trials (RCTs) was established as a mechanism to protect participants against undue harm from research and allow people to recognise any potential risks or benefits associated with the research. A number of interventions have been put forward to improve this process. Outcomes reported in trials of interventions to improve the informed consent process for decisions about trial participation tend to focus on the ‘understanding’ of trial information. However, the operationalization of understanding as a concept, the tools used to measure it and the timing of the measurements are heterogeneous. A lack of clarity exists regarding which outcomes matter (to whom) and why. This inconsistency between studies results in difficulties when making comparisons across studies as evidenced in two recent systematic reviews of informed consent interventions. As such, no optimal method for measuring the impact of these interventions aimed at improving informed consent for RCTs has been identified.

**Methods/Design:**

The project will adopt and adapt methodology previously developed and used in projects developing core outcome sets for assessment of clinical treatments. Specifically, the work will consist of three stages: 1) A systematic methodology review of existing outcome measures of trial informed consent interventions; 2) Interviews with key stakeholders to explore additional outcomes relevant for trial participation decisions; and 3) A Delphi study to refine the core outcome set for evaluation of trial informed consent interventions. All stages will include the stakeholders involved in the various aspects of RCT consent: users (that is, patients), developers (that is, trialists), deliverers (focusing on research nurses) and authorisers (that is, ethics committees). A final consensus meeting including all stakeholders will be held to review outcomes.

**Discussion:**

The ELICIT study aims to develop a core outcome set for the evaluation of interventions intended to improve informed consent for RCTs for use in future RCTs and reviews, thereby improving the reliability and consistency of research in this area.

**Electronic supplementary material:**

The online version of this article (doi:10.1186/s13063-015-1011-8) contains supplementary material, which is available to authorized users.

## Background

The process of obtaining informed consent for participation in randomised controlled trials (RCTs) was established as a mechanism to help protect participants against undue harm from research and allow people to recognise any potential risks or benefits associated with the research [1]. As part of the invitation to take part, currently, potential trial participants are usually provided with an information leaflet about the trial and given the opportunity to have any questions answered by a member of the trial team before indicating their consent by signing agreement to a series of questions [2]. The adequacy of both the indication of consent and the broader processes in which it exists has been called into question.

Much of the research to date investigating the adequacy of the invitation and recruitment process in RCTs (herein referred to as the RCT decision process) has tended to focus on aspects relating to how well informed potential participants are or whether they are recruited into the trial. These current ‘outcomes’ of the RCT decision process, which are fairly ubiquitous in the available evaluation literature [3–8], do not reflect all the features considered important by the various stakeholders in this context [9]. Outcome measures have been selected largely by the researchers who may not have included or consulted consumers and carers (or indeed other stakeholders) about which outcomes they would prioritise [8]. In addition to how informed potential participants are and whether they are recruited into trials, other issues such as decision conflict, decision regret, trust, coercion, resource requirement (time and money) honesty, autonomy and consumer involvement have been identified as potentially important [9]. It is thus important to consider the wider features of RCT decision-making processes (and the interventions that are designed to improve them) and this broader range of implications. Further research is needed to clarify which outcomes matter to whom and why to allow consideration of a broader range of outcomes that are of interest to all relevant decision makers.

As mentioned previously, outcomes reported in trials of interventions targeting decisions about whether to participate in a RCT have, to date, tended to focus on how informed potential participants are (knowledge or understanding of trial information) and recruitment (or accrual or enrolment) [7]. Whilst recruitment and associated concepts are generally operationalised in a standardised manner, the conceptualisation of knowledge or understanding, their operationalisation and the tools used to measure them, and the timing of their measurement are heterogeneous [4, 8]. Indeed, the measurement tools for knowledge or understanding are often study-specific and not well validated [4, 8]. This inconsistency between study measures results in difficulties when making comparisons across studies as demonstrated in two recent systematic reviews of informed consent interventions [4, 8]. Synthesising outcome data that may have been collected using tools of different formats, length, administration and assessment is a significant obstacle when considering how to bring these studies together meaningfully in a meta-analysis. The conclusions of the two reviews that had been previously undertaken are limited by the heterogeneity of the included outcome measures [4, 8].

Core outcome sets aim to define a set of outcomes that should be considered ‘core’ for the evaluation and reporting of specific interventions or conditions (that is, the set of outcomes that should always be considered and ideally measured in any evaluation) [10]. There is a growing body of literature to provide support for development of core outcome sets [10–15]. Specifically, they are developed using consensus methods involving stakeholder groups, such as health professional and patients, so as to ensure that the outcomes being defined are both clinically and personally relevant for the individuals involved [10, 14]. Generation of a core outcome set is not expected to be mutually exclusive to the measurement of other outcomes. However, a core set will foster greater consistency in outcome reporting between studies and lead to more meaningful data being available to contribute to meta-analysis [10, 14]. Moreover, core outcome sets can minimise the threat of outcome reporting bias by ensuring consistency between what is measured and what is reported [10, 14]. Ultimately, they should improve the overall efficiency and quality of the evidence on which healthcare decisions can be made [10, 14].

To date the majority of core outcome sets have been developed for the evaluation of interventions in clinical conditions. They identify both patient-reported and clinical outcomes as core for particular conditions or interventions [11–14]. This project is different in that whilst it aims to develop a core outcome set using established methodology, the interventions of interest are more methodological than clinical. The core set will be for the evaluation of interventions intended to improve the processes by which potential participants are invited to participate and (particularly) supported to make decisions about whether to participate in RCTs. (This set of interventions encompasses informed consent procedures as a subset). Details of this project have been registered and included in the Core Outcome Measures in Effectiveness Trials (COMET) initiative database [15].

### Aims and objectives

#### Aim

The aim of this study is to develop a core outcome set for the evaluation of interventions that aim to improve how people make decisions about whether to participate in RCTs (of healthcare interventions). The scope is restricted to interventions that target the decisions of adults deemed to have adequate mental capacity (as defined in the Adults with Incapacity (Scotland) Act 2000 [16], that is, those persons (over 16) who are incapable of acting, making decisions, communicating decisions, understanding decisions, or retaining the memory of decisions) and who are deciding, prospectively, for themselves about participation in a definitive randomised trial evaluating the effectiveness of healthcare interventions. In addition to its role as a core outcome set to evaluate interventions, the set could also be used as a way to evaluate the processes used to invite people to take part.

#### Objectives

The specific study objectives are:

1. To identify a list of outcomes either previously reported in studies evaluating interventions aiming to improve how people decide about whether to participate in RCTs, suggested as relevant in articles discussing the issue of decisions about participation in an RCT or articles looking critically at the issue of informed consent more generally (in research) that can be seen to have potential relevance to trial contexts.
2. To explore additional outcomes relevant for interventions aiming to improve how people decide whether to participate in an RCT using semi-structured interviews with stakeholders.
3. To define a core outcome set for evaluation of interventions to improve decisions about trial participation through a modified Delphi survey and consensus group meeting.

## Methods/Design

### Systematic review

#### Criteria for consideration of included studies

##### Types of studies

Included studies will have attempted to identify or investigate what is important in the processes used to help adults (with capacity) decide whether or not to participate in randomised clinical trials. For the purposes of this study, the focus will be on decisions to participate in so-called ‘effectiveness’ RCTs (that is, ‘studies (also known as pragmatic studies) that examine interventions under circumstances that more closely approach real-world practice, with more heterogeneous patient populations, less-standardized treatment protocols, and delivery in routine clinical settings’ [17]). The kinds of studies that have attempted to identify or investigate what is important may include both experimental study designs and exploratory studies. The experimental studies will have assessed the effects of interventions intended to standardise or improve (aspects of) processes recruiting people to trials. Studies will include literature reviews with/without meta-analyses, randomised controlled trials, controlled trials, case series and prospective cohorts. Specifically, the nature of intervention, findings or discussion (in the included study) must focus on a plausible mechanism of action which impacts on an aspect that could be considered important for the decision to participate. Exploratory studies (using observations, interviews, focus groups and other methods) that have explored aspects of the RCT decision process (for adults with capacity) will also be included. This literature will be supplemented with a critical analysis of articles that have discussed the ethical aspects of the processes of recruiting people to research, and any potentially relevant outcomes for trial participation decisions suggested will also be included.

##### Exclusion criteria

Papers or articles (both experimental and exploratory) that consider the decision to participate in research studies that are not definitive effectiveness RCTs will be excluded.

#### Search methods for identification of studies

A search strategy has been designed by the Senior Information Scientist (CF), refined through discussion with the Chief Investigator (KG) and informed by previous work conducted in this area. The search for experimental studies will focus on interventions targeting the decision to participate but will be supplemented with a systematic review of interventions that aim to improve recruitment. The purpose of this is to be able to include studies whose primary aim may not be to improve the decision to participate but may have still measured or considered outcomes of relevance where there is a plausible mechanism that impacts an aspect that could be considered important for the decision to participate, for example, an open trial design. Specific search strategies have been designed to capture the experimental studies and exploratory studies separately. The exploratory searches will exclude the records retrieved by the experimental search to avoid duplication of the results. Searches will be applied to MEDLINE (from 1946), EMBASE (from 1947) and CINAHL (from 1981) to the present for both sets of literature, and in addition, CENTRAL and the Cochrane Methodology Register will be searched for experimental studies. A formal search strategy to identify the discursive literature on the ethical aspects of the processes of recruiting people to RCTs will be designed. It is likely that some of these articles will also be identified through reference linking of known texts (books rather than journals) and of those articles identified in the quantitative and qualitative search. Detailed search strategies are provided in Additional file 1. The review will be reported in accordance with PRISMA guidelines [18].

A search for additional studies will be undertaken by checking the references of the included studies. Citation searches of the included studies will also be performed using Scopus, Science Citation Index and the Social Science Citation Index. Experts in the area will be contacted (through email and social media) to identify any additional studies of importance.

##### Eligibility of studies

Citations identified through the search will be independently assessed by two reviewers (KG and a second reviewer). Full text papers will be obtained for those studies that on initial screening are considered potentially relevant and will be further assessed for inclusion. Any studies not meeting inclusion criteria will be excluded. The eligible full text papers will be assessed independently by two reviewers with a third reviewer acting as an arbiter is there is any disagreement. Reference lists of all included studies will be examined for further relevant studies.

##### Data extraction

Information from the primary studies will be extracted independently by two reviewers and reviewed to assess agreement that all outcomes have been identified. The following summary data will be extracted and summarised from each study: study type; study aim; author details; year and journal of publication; and where relevant, host study context (for example, condition, trial design and intervention(s)). Specific details on the outcome measures used as indicators of important for the RCT decision and recruitment process will be extracted. These will include rationale provided by study authors for the selection of outcomes used; all outcomes reported (primary, secondary and others); whether the primary outcome is clearly defined (to allow reproducibility and to include timing of measurement (time point or period), person collecting/measuring outcome, and how outcome was operationalised or measured (for example, validated tool/measure)); and how the outcome was reported. Where possible, the same data will be extracted from the included qualitative studies and additional articles that discuss outcomes for decisions about RCT participation. It may be that more discursive papers focus on the conceptualisation and operationalization of potential outcomes and/or measures (whether previously measured in this context or not), and these will be extracted and reported as such. The study authors will be contacted if published data are unavailable or unclear.

##### Data analysis

Data will be summarised and presented in tabular form. Outcomes will be grouped into outcome domains following data abstraction and will likely include (but not be restricted to) areas such as understanding, satisfaction, anxiety, decision making, trust etcetera. Broad outcome domains will be determined through discussion with the project team and reviewed by the Advisory Group to assess the suitability of the domain name and grouping of outcomes. A framework incorporating the outcome domains will be developed, following previously published examples [12], to aid in analysis.

Outcomes identified as being assessed with tools (whether self-reported or not) will be reviewed and grouped into outcome domains in a similar process to that previously described. Specifically, we will extract items verbatim from the identified tools. We will consider the meaning (possible interpretations of) of these items and compare them for convergence (similarity) and divergence (difference). Duplicate and very closely similar items will be removed to create a reduced item set. The reduced item set will then be systematically categorised into outcome domains according to the concept they address for example, understanding, satisfaction, anxiety, decision making, trust and domains still to be determined/confirmed. All individual items will be mapped to at least one relevant domain. This iterative process will be performed by the project advisory group. This is in line with previously published examples [12].

For each broad outcome domain, evaluations of rationales (and consideration of which outcomes they suggest are important), variant outcome conceptualisations and measures used to reflect that domain, the frequency of selection of individual outcomes (highlighting those which have been discussed in the literature but not measured in experimental studies), and the timing of their implementation will be conducted.

### Identification of outcomes of importance to stakeholders

#### Participant identification and recruitment

Approximately thirty expert stakeholders will be invited to participate in semi-structured interviews in which their views on the outcomes identified thus far will be explored. At this stage, the focus will be on stakeholders who have some interest in trial participation, decision making and/or the ethics of recruitment to research. A range of expertise and perspectives will be sought, and will likely include (where all non-patient participants will be providing expert opinion and as much as possible identified from known existing professional networks) the following:

1. Patients (trial experienced versus trial naive) or advocates,
2. Trialists,
3. Research nurses,
4. Social scientists and ethicists with an interest in trials and/or informed consent, and
5. Psychologists with an interest in communication research.

The rationale for including only research nurses in the Delphi as opposed to other clinical staff is based on the premise that research nurses, unlike other clinical staff, are employed primarily to recruit potential participants to research studies. Linked to this, research nurses are often the staff responsible for obtaining informed consent from potential trial participants and continuing the consent process through trial follow-up. As such, they were deemed the most appropriate clinical stakeholder group for inclusion in the Delphi process.

A purposive sample of each stakeholder group will be identified. Sampling will be conducted within each stakeholder group to provide a balance of participants across the groups. To ensure a representative balance, sampling will primarily be informed by the country of residence of the participant (for non-patient participants) and the clinical area or population in which they work. The total of 30 interviews will be made up of approximately six interviews per stakeholder group but will be adjusted according to methods of good practice (see below). Specific organisations, authors of key papers identified in the literature search (in **3** above), and contacts from existing professional networks will be invited. Each group will be identified from the following sources:

1. Patients and advocates from the Scottish Health Research Register (SHARE), James Lind Alliance and from health literacy groups (for example, www.healthliteracy.org.uk).
2. All non-patient groups will first be identified through known professional networks. If additional numbers are required, non-patient participants will be identified as follows:
  - Trialists will be identified through the Society for Clinical Trials and UK Clinical Trials Units.
  - Research nurses will be invited through the Scottish Research Nurse and Coordinators Network (SRNCN), and the NIHR Clinical Research Network and identified through twitter (for example, using the #CRNurse).
  - Social scientists will be identified through the Society for Social Medicine.
  - Ethicists through the Association of Bioethics, Feminist Approaches to Bioethics Network and Society for Applied Philosophy.
  - Psychologists through the Society for Medical Decision Making and the Shared@EACH facebook page.

The qualitative sample of 30 participants is in line with numbers reported for the interview stage of previous Delphi studies and deemed likely to be sufficient for saturation [19, 20]; however, the sample size will be adjusted accordingly using a stopping criterion of three if new opinions continue to emerge from the data (that is, three consecutive interviews with no additional material terminates data collection).

Through purposive sampling, a diverse range of participants will be included. The aim will be to recruit participants with a wide variety of trial experience and diverse perspectives on features that are important to people when deciding whether or not to participate in an RCT.

Prospective participants will be sent an invitation letter and study information in the initial email sent from the gatekeeper of the list serves or social media contacts. Interested parties will be asked to contact the lead researcher (KG) directly to arrange a convenient time to conduct the interview. Before the interview commences, the lead researcher (KG) will obtain informed consent, both written and verbal, from participants. The interviews will be conducted over the phone so as to allow inclusion of participants across a wide geographic area and will last between 20 and 60 minutes. All interviews will be audio-recorded and transcribed for analysis.

### Date collection

A summary of findings from the literature review will be shared in advance. A definition and interpretive notes will be developed by the Advisory Group for each of the outcome domains and associated outcomes. These will be provided to aid participants understanding.

Participants will be asked to comment generally on whether and why particular features are important and how they conceptualise and assess the importance of these outcomes, for example, why potential participants’ knowledge about one aspect of the trials is normatively or practically more significant than knowledge about other aspects (or outcomes other than knowledge). Specifically, informants will be asked to reflect on the applicability of identified outcomes, suggest additional outcomes of relevance and why these outcomes could be important in a trial context and identify which outcomes they would consider core and why. This will allow identification of outcomes that, as yet, have not been considered or measured in empirical studies of interventions to improve the decision-making process for trial participation, or discussed in literature on research ethics. All interviews will be audio-recorded and transcribed.

### Data analysis

The interview analysis will begin during data collection, with the initial data analysis of preliminary interviews being used to inform the subsequent data collection. This process allows development and refinement of key topics for exploration in subsequent interviews. Interview data will be coded and compared through a process of constant comparison [21] to provide a summary of the key points about what stakeholders consider important in this context. Each stakeholder group will be analysed in parallel, but within and across group analysis will be conducted to explore areas of convergence and areas of divergence. The transcripts will be imported into NVivo (a qualitative analysis software, version 10, 2013:QSR International) and analysed using the framework approach, which is an established interpretive approach that uses constant comparison techniques [22, 23]. The framework approach is made up of five stages: familiarisation with the data, development of a thematic framework, indexing data, devising thematic charts, and mapping and interpreting data [23]. The codes identified and the associated data assigned to them from the transcripts will be reviewed by a second member of the project team. Specifically, the analysis will be oriented to address the aim of identifying the range of outcomes that might be considered important and the reasons used to justify assessment of them as important.

Ethical approval for the qualitative interviews with stakeholders has been obtained. This study was approved by the National Research Ethics Service Committee London-Chelsea (REC reference 15/LO/0375).

### Define core outcome set for evaluation of trial informed consent interventions

#### Overview

To identify outcomes of importance across groups involved directly in decisions about participation in RCTs (for example, designers (trialists and lead clinicians), recruiters (research nurses), potential participants and authorisers (ethics committees), a Delphi consensus survey approach, employing up to three rounds of rating, will be used. The use of a Delphi will allow participants responses to be obtained without influence from others and allows analysis based on individual stakeholder groups, giving equal weighting to all participants. An overview of the Delphi exercise is given in Additional file 2.

#### Identification of potential outcomes

The list of potential outcomes generated from the systematic review and interviews will form the basis of the international Delphi survey to refine the items into a core outcome set. The outcomes will be listed individually but also grouped into relevant domains (as in Data analysis in 3.1.2) so as to assist in interpretation and use of the Delphi survey. The outcome list will be reviewed by the Advisory Group (composed of content and methodology experts in the fields of decision making, ethics and clinical trials) specifically for comprehension but also for suitability of the outcome domain structure and groupings.

### Participants

Stakeholders will be invited to participate in the Delphi survey through email distribution lists, social media or direct contact. Potential participants will be contacted by a gatekeeper on behalf of the research team. Each stakeholder group will be identified from the following sources:

1. Patients and advocates from the Scottish Health Research Register (SHARE), James Lind Alliance, and from health literacy groups (for example, www.healthliteracy.org.uk).
2. All non-patient groups will first be identified through known professional networks. If additional numbers are required, non-patient participants will be identified as follows:
  - Trialists will be identified through the Society for Clinical Trials and UK Clinical Trials Units.
  - Research nurses will be invited through the Scottish Research Nurse and Coordinators Network (SRNCN), and the NIHR Clinical Research Network and identified through twitter (for example, using the #CRNurse).
  - Social scientists will be identified through the Society for Social Medicine.
  - Ethicists through the Association of Bioethics, Feminist Approaches to Bioethics Network and Society for Applied Philosophy.
  - Psychologists through the Society for Medical Decision Making and the Shared@EACH facebook page.
  - Ethics committee chairs through the National Research Ethics Service and known contacts at Institutional Research Boards (IRBs).

Justifications for sample size included in this survey are discussed below in section 5.7. Invitation letters describing the Delphi survey will be sent through the email distribution list with interested parties being asked to complete the Delphi questionnaire, which will be an online questionnaire accessed through an embedded web link in a direct email.

The number of participants completing the questionnaire will be recorded, and this will be compared to completion throughout subsequent rounds of the Delphi to assess attrition. In addition, the computer software will record who accesses the Delphi website but does not go on to complete the scoring.

The opening page for the Delphi survey will provide a brief introduction of the aim of the study and will remind participants of the importance of completing all rounds of the process. Reminder emails will be sent to non-responders at each round. Participants who agree to participate will be asked to register their details online (including specifying which stakeholder group they are associated), which will generate a unique identifier against which their data will be stored and reminder emails for non-response will be generated. Participants will not be able to identify other participants or others individual responses. Explicit consent will not be sought for the Delphi survey. Instead, consent will be implicit by completion and return of the questionnaire. However, the questionnaire will include a section to state that participants are happy to be contacted again in the future for additional research activities linked to this stage, that is, the final discussion group.

Ethical approval for the Delphi survey with stakeholders has been obtained.

### Delphi survey

#### Delphi round 1

The survey will be developed into a web-based application using software (known as COMET Delphi Manager) developed by Programmers with experience of developing online Delphi surveys and who work closely with the COMET Initiative (www.comet-initiative.org/). A bespoke website (modified from existing templates) and unique web link will be created. During the first round of the online questionnaire, the participant’s name and email address will be requested. This information will be stored in a separate database and used to generate the unique identifier. The first question will ask each respondent to identify their stakeholder group(s) and to specify their experience of RCTs. Participants will be asked to complete each round of the Delphi survey within 3 weeks of receiving the email for each round. A reminder email will be sent to non-responders at the end of week 2 to prompt their completion of the survey.

#### Round 1 survey format

The survey will be presented through an online platform, as described above. A definition (as determined by the Advisory Group) of each of the outcome domains and associated outcomes will be provided to aid participant understanding. Round 1 content will include a list of outcomes for scoring that is ordered alphabetically by domain and a free text box to allow participants to add any additional outcomes and provide an associated score. The respondents will be asked to consider the following:

*Think about the process of being invited to take part in a clinical trial. How important do you think each item listed below would be in judging how well that process had been conducted?*

As defined in other Delphi surveys for developing core outcome sets, participants will be asked to score each of the listed items using the Grading of Recommendations, Assessment, Development and Evaluations (GRADE) scale of 1 to 9 [11, 13, 23]. The scale will be annotated to illustrate that a score of 1 to 3 is interpreted as having ‘limited importance’, 4 to 6 as ‘important but not critical’ and 7 to 9 as ‘critical’ [23]. As outlined above, a free text box will be provided for participants to include any additional outcomes and associated scores.

#### Analysis of round 1

Descriptive statistics will be used to summarise the results from each round. For each outcome, the distribution of scores (frequency distribution, the median and interquartile range) will be summarised by stakeholder group alongside the total number of participants who scored the outcome. Any new additional outcomes listed by participants in round 1 will be reviewed and coded by two members of the study team to ensure they are independent from those listed, with a third reviewer acting as an arbiter if there is disagreement. Participants will be instructed to rate all outcomes on their own merit even if they appear similar. All outcomes will be carried forward to round 2.

#### Response rate in round 1

Following the completion of round 1, the results will be presented for the research team as the total number of registrations to the website, the number of participants who have completed round 1, the total number of participants in each stakeholder group and the number of respondents as a percentage of those invited by stakeholder group.

Round 2 assumes sufficient numbers respond to round 1 across each of the stakeholder groups. If there are inadequate numbers for one or more stakeholder groups, the Advisory Group will be consulted. For the purposes of this project, inadequate numbers have been predefined by the project team as fewer than 20 in any stakeholder group. The rationale for this lower limit has been informed by a recent study by Harman et al. who used a lower limit of 10 in any stakeholder group [11]. As we are randomising participants to one of two ways of receiving feedback, the lower limit for the ELICIT study is 20. Those who have not taken part in round 1 (that is, did not score outcomes) will not be invited to participate in further rounds.

#### Delphi round 2

Round 2 of the Delphi survey will also be presented online. During Round 2, participants will be randomised to one of two groups for the type of feedback they receive, as conducted in previous core outcome set projects [12, 13]. The first group will receive feedback at the level of their own individual stakeholder group, whereas the second group will receive feedback from each of the stakeholder groups. Participants will be presented with feedback (number of respondents and distribution of scores across the 1-9 scale) relevant for their particular randomised group and personalised feedback relating to their previous score for each outcome. Participants will be asked to consider their score within the context of the scores of others in their randomised group and then rescore the outcome again, using the nine- point scale.

#### Analysis of round 2

The total number of participants invited to participate in round 2 will be recorded and compared to the total number of round 2 responders (and compared for response bias between the two randomised feedback groups). For each outcome, the distribution of scores will be summarised across stakeholder groups. All outcomes will be carried forward to round 3.

#### Delphi round 3

In the final online round of the Delphi, participants will be presented with the distribution of scores for each outcome for each of the stakeholder groups and reminded of their personal score from Round 2. Participants will then be asked to rescore all outcomes and consider whether, and why, they should be included in a core outcome set.

#### Analysis of round 3

The total number of participants invited to round 3 will be recorded and compared to the total number of round 3 responders. For the final analysis, for each outcome, the number of participants who scored the item and the distribution of scores will be summarised, alongside the number of respondents who scored the items across all three rounds. For each outcome, the proportion of respondents scoring 1 to 3, 4 to 6 and 7 to 9 on the Likert scale will be calculated for each item. Each outcome will be classified as: ‘consensus in’ (that is, consensus that the outcome should be included in a core set), ‘consensus out’ (that is, consensus that the outcome should not be included in a core set) or ‘no consensus’ (that is, items that are equivocal and require further research for clarification), according to the classifications in Table 1.

**Table 1:** Definition of consensus

Consensus classification	Description	Definition
*Consensus in*	Consensus that outcome should be included in the core outcome set	≥70 % scoring 7 to 9 AND <15 % scoring 1 to 3
*Consensus out*	Consensus that outcome should not be included in the core outcome set	≥70 % scoring 1 to 3 AND <15 % scoring 7 to 9
*No consensus*	Uncertainty about importance of outcome	Anything else and no new compelling reasons in the comments boxes regarding why.

(taken from Harman et al. 2013 and Waters et al. 2014 [10, 12])

Justification for these levels and definition of consensus are given below in section 5.6.

### Consensus meeting

The final phase of the consensus study will be a face-to-face meeting with key participants from the Delphi exercise and preliminary interviews. The main aim of the consensus meeting will be to determine consensus (in or out) for those items that exhibited no consensus.

Potential participants will have been identified through earlier rounds of the research and will have consented to be contacted again for additional stages of the project. Informed consent will be obtained (by the lead researcher) from participants before initiation of the discussion group. The discussion group will be face-to-face and will be facilitated by the research team. The discussion will be conducted at a location agreed-upon as mutually convenient by most participants and will likely last up to 3 hours. The group discussion will be audio-recorded and transcribed for analysis.

The results from the Delphi survey will be presented, with a focus for discussions being those outcomes for which there was disagreement in Round 3 and to further validate and agree on a final list of outcomes that will constitute the ‘core outcome set’. The final format of the consensus meeting will be determined based on a review of experiences from similar projects and the percentage agreement between the group and stakeholder overall scores, which will help to identify areas of divergence or significant agreement. We will document the discussions during the consensus meeting paying particular attention to any gaps or minority concerns about the list that achieved consensus so as to reflect on any potential limitations. These discussions will also provide further opportunity to hear a refined discussion of the reasoning for regarding some outcome domains as important. By the end of the consensus meeting, ‘what’ outcomes to measure should have been identified. Questions about ‘how’ to measure them (that is, which instruments to use) may still require further work, but this will not preclude the inclusion of any outcome (whether currently measurable or not) in the final recommended core outcome set. Determining ‘how’ to measure the identified outcome is outside the scope of this study.

### Justification for definition of consensus

With reference to the Delphi survey, the definition of consensus used in the ELICIT study to determine inclusion of an outcome will be based on previously published examples [11, 13]. These definitions (see Table 1) are built on the premise that an outcome should be included in the core set if the majority agree on its critical importance and only a minority consider it unimportant. Conversely, for exclusion of an outcome, the majority must agree on its lack of importance with only a minority considering it critical. The decision regarding thresholds for inclusion and exclusion in Delphi studies varies, with no defined rules on limits [10–14]. However, although subjective, the levels described in the ELICIT study have been implemented in other Delphi studies to define core outcome sets [10–14]. To minimise bias, the definition of consensus should always be specified at study outset and not post hoc based on preferences of the study team.

### Statistical considerations

There is currently no standard method for determining sample size calculations for Delphi studies. However, there is emerging evidence in the literature that expert panels of around 20 can provide stable results [24]. As such, a pragmatic approach is adopted taking into consideration the access to participants and interest from those invited, manageability of data and representativeness. Through discussion with the project team, the minimum sample size required for analysis was determined to be 20 participants per stakeholder group. Therefore, to allow for 50 % attrition between the three rounds, a minimum of 80 participants per stakeholder group will be invited to participate in the Delphi survey; however, efforts will be made to maximise response rates across stakeholder groups so as to ensure that attrition bias is minimal.

## Discussion

To date, no core outcome set for the evaluation of interventions for improving processes of inviting people to participate in RCTs has been published. The development of a core outcome set in this methodological area aims to improve the conduct, interpretation and comparison of past and future studies by minimising heterogeneity across studies and reducing the potential risk of outcome selection and reporting bias in studies of this type. Determining which outcomes to measure for evaluation of RCT recruitment processes and interventions will also require further reflections on how to measure these core outcomes and will provide key areas for future research. The ELICIT study will involve multiple key stakeholders through several stages of the study to ensure that any core outcome set defined is fit for purpose and well accepted in future research, both in the UK and internationally.

## Trial status

Overall, the protocol does not report a trial but rather development of a core outcome set for use in future trials of interventions to improve informed consent. However, there is a randomised component to the Delphi survey (see section 5.4.2a). This project is currently in the set-up phase.

## Additional files

Additional file 1:
**Search strategies.** Search strategies for each of the individual platforms searched. (DOCX 23 kb) Additional file 2:
**Delphi Overview.** Figure providing an overview of the Delphi process. (DOCX 32 kb)

## Abbreviations

COMET: Core Outcome Measures in Effectiveness Trials
ELICIT: Evaluation of Interventions for informed consent for randomised controlled trials
GRADE: Grading of Recommendations, Assessment, Development and Evaluations
IRB: Institutional Research Boards
NIHR: National Institute for Health Research
RCT: randomised controlled trial
REC: Research Ethics Committee
SHARE: Scottish Health Research Register
SRNCN: Scottish Research Nurse and Coordinators Network
