# Supplementary material for: Evaluation of interventions for informed consent for randomised controlled trials (ELICIT): protocol for a systematic review of the literature and identification of a core outcome set using a Delphi survey
Source: Trials. 2015 Oct 27;16:484. doi: 10.1186/s13063-015-1011-8 (PMC4624669; doi:10.1186/s13063-015-1011-8)
Supplement: Additional file 1: — Search strategies. Search strategies for each of the individual platforms searched. (DOCX 23 kb) [file 13063_2015_1011_MOESM1_ESM.docx]

**Additional File 1**

**Search Strategies**

***Evaluative studies***

**Embase 2014 1947 - Week 22; Ovid MEDLINE(R) 1946 - May Week 3 2014; Ovid MEDLINE(R) In-Process & Other Non-Indexed Citations June 02, 2014.**

***Ovid Multifile Search URL: https://shibboleth.ovid.com/***

1 exp *informed consent/ (31540)

2 (informed adj3 (consent or decision? or choice)).tw. (77378)

3 (consent$ adj2 (process or form$ or document$ or written or verbal)).tw. (20821)

4 *patient participation/ (15773)

5 Refusal to participate/ (1285)

6 or/1-5 (117104)

7 exp clinical trials as topic/ use mesz (280332)

8 exp "clinical trial (topic)"/ use emcz (104525)

9 research subjects/ use mesz (5115)

10 research subject/ use emcz (5333)

11 Biomedical Research/ use mesz (35611)

12 ((research or trial?) adj3 (subject? or participant?)).tw. (56726)

13 exp medical research/ use emcz (311838)

14 or/7-13 (773516)

15 6 and 14 (12659)

16 ((consent$ or information or particpa$ or tak$ part or enrol$ or recruit$ or enter$) adj5 trial?).tw. (58984)

17 15 or 16 (69696)

18 *Information Dissemination/ (8407)

19 *patient education as topic/ use mesz (30955)

20 *patient education/ use emcz (23901)

21 *interviews as topic/ use mesz (3494)

22 exp *interview/ use emcz (7452)

23 *communication/ use mesz (26640)

24 *interpersonal communication/ use emcz (37211)

25 *audiovisual aids/ use mesz (3554)

26 *audiovisual aid/ use emcz (74)

27 *computer-assisted instruction/ use mesz (7042)

28 *decision support techniques/ use mesz (6685)

29 *decision support system/ use emcz (6654)

30 *patient decision making/ use emcz (942)

31 *social support/ (31213)

32 or/18-31 (189720)

33 (information adj3 (present$ or communicat$ or explain$ or disseminat$)).tw. (49376)

34 (visual aid? or decision aid? or diagram? or video? or graph?).tw. (216168)

35 (audiovisual or videotape?).tw. (26816)

36 (readability or literacy).tw. (21038)

37 (information adj3 format?).tw. (1053)

38 (information adj3 (leaflet? or booklet? or pamphlet? or sheet? or written)).tw. (9476)

39 ((interactive or communicat$) adj3 tool?).tw. (4960)

40 (interactive adj3 communicat$).tw. (745)

41 (consent adj3 (enhanc$ or improv$ or increas$)).tw. (1266)

42 (consent adj3 (method? or procedure? or obtain$ or elicit$)).tw. (22276)

43 or/32-42 (517400)

44 17 and 43 (5071)

45 randomized controlled trial.pt. (374165)

46 controlled clinical trial.pt. (88395)

47 randomi?ed.ab. (809919)

48 exp clinical trial/ use emcz (976729)

49 major clinical study/ use emcz (2221283)

50 randomization/ use emcz (62356)

51 placebo.ab. (352205)

52 drug therapy.fs. (1699822)

53 randomly.ab. (478383)

54 trial.ab. (708326)

55 groups.ab. (3160216)

56 or/45-55 (7998337)

57 (chang$ or evaluat$).tw. (9334222)

58 comparative study/ (2323078)

59 (compare$ or compara$).tw. (6533203)

60 (cohort$ or case series).tw. (753015)

61 (prospective$ or retrospective$).tw. (1894828)

62 comparison.tw. (1647556)

63 or/56-62 (19540051)

64 44 and 63 (4019)

65 remove duplicates from 64 (2965)

**CINAHL (1981 – 5^th^ June 2014)**

***URL: www.ebscohost.com/***

S1 (MH "Consent (Research)") OR TX ( (informed N3 (consent or decision* or choice)) ) OR TX ( (consent* N2 (process or form* or document* or written or verbal)) ) 9,893

S2 (MH "Research Subject Recruitment") 7,561

S3 (MM "Consumer Participation") 5,625

S4 (MH "Refusal to Participate") 54

S5 S1 OR S2 OR S3 OR S4 22,370

S6 (MH "Clinical Research") OR (MH "Health Services Research+") 19,638

S7 (MH "Research+") 1,007,304

S8 (MH "Clinical Research+") 9,128

S9 TX ((research or trial*) N3 (subject* or participant*)) 20,817

S10 S6 OR S7 OR S8 OR S9 1,008,496

S11 S5 AND S10 15,947

S12 ((consent* or information or particpa* or tak* part or enrol* or recruit* or enter*) N5 trial*) 4,909

S13 S11 OR S12 20,199

S14 (MM "Emotional Support (Iowa NIC)") 1

S15 (MM "Social Support (Iowa NOC)") 1

S16 (MM "Decision Support Techniques") 823

S17 (MM "Computer Assisted Instruction") 3,245

S18 (MM "Audiovisuals") 545

S19 (MM "Communication+") 58,418

S20 (MM "Interviews+") 1,301

S21 (MM "Patient Education") 13,488

S22 TX ( (consent N3 (enhanc* or improv* or increas*)) ) OR TX ( (consent N3 (method* or procedure* or obtain* or elicit*)) ) 1,898

S23 TX ( ((interactive or communicat*) N3 tool*) ) OR TX (interactive N3 communicat*) 1,082

S24 TX ( (readability or literacy) ) OR TX (information N3 format*) OR TX ( (information N3 (leaflet* or booklet* or pamphlet* or sheet* or written)) ) 11,776

S25 TX ( (information N3 (present* or communicat* or explain* or disseminat*)) ) OR TX ( (visual aid* or decision aid* or diagram* or video* or graph*) ) OR TX ( (audiovisual or videotape*) ) 37,819

S26 S14 OR S15 OR S16 OR S17 OR S18 OR S19 OR S20 OR S21 OR S22 OR S23 OR S24 OR S25 118,665

S27 S13 AND S26 2,940

**Cochrane Library (CMR Issue 3 2012; CENTRAL Issue 5 2014)**

**URL:** [**http://www3.interscience.wiley.com/**](http://www3.interscience.wiley.com/)

#1 MeSH descriptor: [Informed Consent] explode all trees

#2 (informed near/3 (consent or decision or choice)):ti,ab,kw (Word variations have been searched)

#3 (consent near/2 (process or form or document or written or verbal)):ti,ab,kw (Word variations have been searched)

#4 MeSH descriptor: [Patient Participation] explode all trees

#5 MeSH descriptor: [Refusal to Participate] this term only

#6 #1 or #2 or #3 or #4 or #5

#7 MeSH descriptor: [Clinical Trials as Topic] explode all trees

#8 MeSH descriptor: [Research Subjects] this term only

#9 MeSH descriptor: [Biomedical Research] explode all trees

#10 ((research or trial) near/3 (subject or participant))

#11 #7 or #8 or #9 or #10

#12 #6 and #11

#13 ((consent or information or particpate or take part or enrol or recruit or enter) near/5 trial)

#14 #12 or #13

#15 MeSH descriptor: [Information Dissemination] this term only

#16 MeSH descriptor: [Patient Education as Topic] explode all trees

#17 MeSH descriptor: [Interviews as Topic] this term only

#18 MeSH descriptor: [Communication] this term only

#19 MeSH descriptor: [Audiovisual Aids] this term only

#20 MeSH descriptor: [Computer-Assisted Instruction] this term only

#21 MeSH descriptor: [Decision Support Techniques] this term only

#22 MeSH descriptor: [Social Support] this term only

#23 (information near/3 (present or communicate or explain or disseminate)):ti,ab,kw (Word variations have been searched)

#24 (visual aid or decision aid or diagram or video or graph):ti,ab,kw (Word variations have been searched)

#25 (audiovisual or videotape):ti,ab,kw (Word variations have been searched)

#26 (readability or literacy):ti,ab,kw (Word variations have been searched)

#27 (information near/3 format):ti,ab,kw (Word variations have been searched)

#28 (information near/3 (leaflet or booklet or pamphlet or sheet or written)):ti,ab,kw (Word variations have been searched)

#29 ((interactive or communicate) near/3 tool):ti,ab,kw (Word variations have been searched)

#30 (interactive near/3 communicate):ti,ab,kw (Word variations have been searched)

#31 (consent near/3 (enhance or improve or increase)):ti,ab,kw (Word variations have been searched)

#32 (consent near/3 (method or procedure or obtain or elicit)):ti,ab,kw (Word variations have been searched)

#33 #15 or #16 or #17 or #18 or #19 or #20 or #21 or #22 or #23 or #24 or #25 or #26 or #27 or #28 or #29 or #30 or #31 or #32

#34 #14 and #33 (948)

***Exploratory studies***

**Embase 1947 to 2014 Week 24; Ovid MEDLINE(R) 1946 to June Week 2 2014; Ovid MEDLINE(R) In-Process & Other Non-Indexed Citations June19th, 2014**

***Ovid Multifile Search URL: https://shibboleth.ovid.com/***

1 exp *informed consent/ (31660)

2 (informed adj3 (consent or decision? or choice)).tw. (75365)

3 (consent$ adj2 (process or form$ or document$ or written or verbal)).tw. (20220)

4 *patient participation/ (15850)

5 Refusal to participate/ (1302)

6 or/1-5 (115012)

7 exp clinical trials as topic/ use mesz (281504)

8 exp "clinical trial (topic)"/ use emcz (106123)

9 research subjects/ use mesz (5138)

10 research subject/ use emcz (5347)

11 Biomedical Research/ use mesz (35887)

12 exp medical research/ use emcz (312362)

13 or/7-12 (775124)

14 6 and 13 (12650)

15 ((consent$ or information or particpa$ or tak$ part or enrol$ or recruit$ or enter$) adj3 trial?).tw. (57535)

16 14 or 15 (41689)

17 qualitative research/ (45499)

18 qualitative analysis/ use emcz (34067)

19 questionnaires/ use mesz (301452)

20 exp questionnaire/ use emcz (402701)

21 exp interviews as topic/ use mesz (55810)

22 exp interview/ use emcz (154682)

23 (qualitative or interview$ or focus group? or questionnaire$ or survey$).tw. (1948498)

24 (ethno$ or grounded or thematic or interpretive or narrative).tw. (98574)

25 or/17-24 (2229681)

26 25 and 16 (5489) [excluding records retrieved from Quantitative Search (2845)]

**CINAHL (1981 – 19th June 2014)**

**URL: www.ebscohost.com/**

S1 (MH "Consent (Research)") OR TX ( (informed N3 (consent or decision* or choice)) ) OR TX ( (consent* N2 (process or form* or document* or written or verbal)) )

S2(MM "Consumer Participation")

S3 (MH "Refusal to Participate")

S4 (MH "Research Subject Recruitment")

S5 S1 OR S2 OR S3 OR S4

S6 (MH "Clinical Research") OR (MH "Health Services Research+")

S7 (MH "Research+")

S8 (MH "Clinical Research+")

S9 S6 OR S7 OR S8

S10 S5 AND S9

S11 TX ((consent* or information or particpa* or tak* part or enrol* or recruit* or enter*) N3 trial*) S12 S10 OR S11

S13 (MH "Qualitative Studies+")

S14 TX( qualitative or interview$ or focus group* or questionnaire$ or survey*).

S15 TX (ethno* or grounded or thematic or interpretive or narrative)

S16 S13 OR S14 OR S15

S17 S12 AND S16 Limiters - Exclude MEDLINE records (876)

**Philosophers Index - December 2014**

S10 S5 AND S9 (492)

S9 S6 OR S7 OR S8

S8 (ZU "clinical research")

S7 (ZU "research") or (ZU "research ethics")

S6 (ZU "medical research") or (ZU "health research")

S5 S1 OR S2 OR S3 OR S4

S4 (consent* N2 (process or form* or document* or written or verbal))

S3 TX (informed N3 (consent or decision* or choice))

S2 (ZU "consent")

S1 (ZU "informed choice") or (ZU "informed consent")
