# Supplementary material for: Evaluation of interventions for informed consent for randomised controlled trials (ELICIT): protocol for a systematic review of the literature and identification of a core outcome set using a Delphi survey
Source: Trials. 2015 Oct 27;16:484. doi: 10.1186/s13063-015-1011-8 (PMC4624669; doi:10.1186/s13063-015-1011-8)
Supplement: Additional file 2: — Delphi Overview. Figure providing an overview of the Delphi process. (DOCX 32 kb) [file 13063_2015_1011_MOESM2_ESM.docx]

**Additional File 2 - Delphi Overview**

Consensus Meeting

Final consensus meeting to determine agreement on equivocal items and define the core outcome set.

Round 3

Feedback is provided showing responses of all stakeholder groups and providing personal scores. Participants are asked to re-score all outcomes and consider which items should be considered core. Analysis to determine consensus.

Round 2

Feedback is provided by stakeholder group. Participants are randomised to feedback (individual stakeholder groups vs. all stakeholder groups) and asked to re-score all outcomes and consider which items should be considered core. Analysis. Inclusion of all items in Round 3.

Potential outcome list identified through systematic review

Outcome list discussed with Advisory Group and developed in to conceptual framework of outcome domains

Invitation sent to (through direct contact, listservs and social media) stakeholders to participate in interviews

Interview participants provided with list of potential outcomes and asked to consider relevance and identify any additional outcomes of importance

Interview data analysed and combined with results from systematic review to generate items for inclusion in the Delphi

Email invitation sent to (through direct contact,listservs and social media) stakeholders to participate in Delphi survey, which includes link to the Delphi and initial registration.

Participants are allocated a unique identifier and required to identify their stakeholder group.

Round 1

Participants are asked to rate the list of outcomes and add, and score, any outcomes they perceive as important but not included. Analysis. Inclusion of all outcomes in to Round 2.
